# Soluble Epoxide Hydrolase Inhibition in Liver Diseases: A Review of Current Research and Knowledge Gaps

**DOI:** 10.3390/biology9060124

**Published:** 2020-06-12

**Authors:** Jeffrey Warner, Josiah Hardesty, Kara Zirnheld, Craig McClain, Dennis Warner, Irina Kirpich

**Affiliations:** 1 Division of Gastroenterology, Hepatology and Nutrition, Department of Medicine, University of Louisville, Louisville, KY 40292, USA; jbwarn01@louisville.edu (J.W.); josiah.hardesty@louisville.edu (J.H.); kara.zirnheld@louisville.edu (K.Z.); craig.mcclain@louisville.edu (C.M.); dennis.warner@louisville.edu (D.W.); 2 Department of Pharmacology and Toxicology, University of Louisville School of Medicine, Louisville, KY 40202, USA; 3 University of Louisville Alcohol Research Center, Department of Medicine, University of Louisville School of Medicine, Louisville, KY 40202, USA; 4 University of Louisville Hepatobiology & Toxicology Center, University of Louisville School of Medicine, Louisville, KY 40202, USA; 5 Robley Rex Veterans Medical Center, Louisville, KY 40206, USA

**Keywords:** non-alcoholic liver disease, metabolic syndrome, fibrosis, portal hypertension, soluble epoxide hydrolase, eicosanoids

## Abstract

Emerging evidence suggests that soluble epoxide hydrolase (sEH) inhibition is a valuable therapeutic strategy for the treatment of numerous diseases, including those of the liver. sEH rapidly degrades cytochrome P450-produced epoxygenated lipids (epoxy-fatty acids), which are synthesized from omega-3 and omega-6 polyunsaturated fatty acids, that generally exert beneficial effects on several cellular processes. sEH hydrolysis of epoxy-fatty acids produces dihydroxy-fatty acids which are typically less biologically active than their parent epoxide. Efforts to develop sEH inhibitors have made available numerous compounds that show therapeutic efficacy and a wide margin of safety in a variety of different diseases, including non-alcoholic fatty liver disease, liver fibrosis, portal hypertension, and others. This review summarizes research efforts which characterize the applications, underlying effects, and molecular mechanisms of sEH inhibitors in these liver diseases and identifies gaps in knowledge for future research.

## 1. Introduction

**Figure 1:**
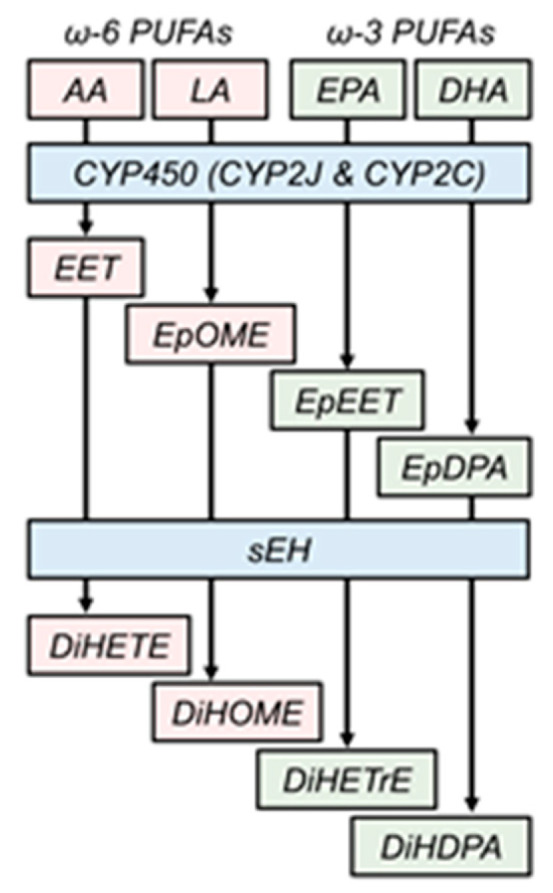
Endogenous role of soluble epoxide hydrolase. Cytochrome P450 monooxygenases catalyze the addition of an epoxide ring to polyunsaturated fatty acids, including omega-6 arachidonic and linoleic acids (AA and LA, respectively) and omega-3 eicosapentaenoic and docosahexaenoic acids (EPA and DHA, respectively) to form epoxy-fatty acids (epFAs). Soluble epoxide hydrolase (sEH) rapidly degrades these beneficial epFAs to their inactive, less active, or deleterious cognate dihydroxylated fatty acids. EET, epoxyeicosatrienoic acid; EpOME, epoxyoctadecenoic acid; EpEET, epoxyeicosatetraenoic acid; EpDPA, epoxydocosapentaenoic acid; DiHETE, dihydroxyeicosatrienoic acid; DiHOME, dihydroxyoctadecenoic acid; DiHETrE, dihydroxyeicosatetraenoic acid; DiHDPA, dihydoxydocosapentaenoic acid.

Since their discovery in the 1970s, mammalian epoxide hydrolases (EHs) have become an increasingly popular focus of research due to their role in disease pathology [1,2]. This family of proteins consists of four enzymes encoded by genes *Ephx1*–*4*, where *Ephx1* and *Ephx2* encode microsomal and soluble EHs (mEH and sEH, respectively), and *Ephx3* and *Ephx4* encode EH3 and EH4, which are not well characterized. EHs are expressed across all domains of life, including both mammalian and non-mammalian animals [3], including insects, frogs, fish, nematodes [4], and plants, protists [5], fungi, and several phyla of bacteria (e.g., actinobacteria, proteobacteria, firmicutes, and others [6]). EHs catalyze the hydrolysis of epoxides by the addition of water to form vicinal diols. Importantly, substrate specificity differs between EHs: mEH generally prefers toxic xenobiotic epoxides, whereas sEH generally prefers endogenous lipid epoxides [1]. An important class of sEH substrates are the cytochrome P450 (CYP450) monooxygenase products of omega-3 and omega-6 polyunsaturated fatty acids (PUFAs), herein referred to as epoxy-fatty acids (epFAs). This class includes epoxyeicosatrienoic acids (EETs, products of arachidonic acid [AA]), epoxyoctadecenoic acids (EpOMEs, products of linoleic acid [LA]), epoxyeicosatetraenoic acids (EpEETs or EEQs, products of eicosapentaenoic acid [EPA]), and epoxydocosapentaenoic acids (EpDPAs or EDPs, products of docosahexaenoic acid [DHA]) (Figure 1). The CYP2C and CYP2J subfamilies are primarily responsible for the production of these epFAs [7], which generally exert beneficial effects on a number of cellular processes. For example, epFAs can be anti-inflammatory [8,9,10] and anti-fibrotic [11], and can promote the resolution of inflammation [12] and tissue regeneration [13]. However, not all epFAs are beneficial—for example, LA (an omega-6 fatty acid)-derived 9,10-EpOME and 12,13-EpOME are associated with respiratory distress and interfere with neutrophil function following infection [14].

**Figure 2:**
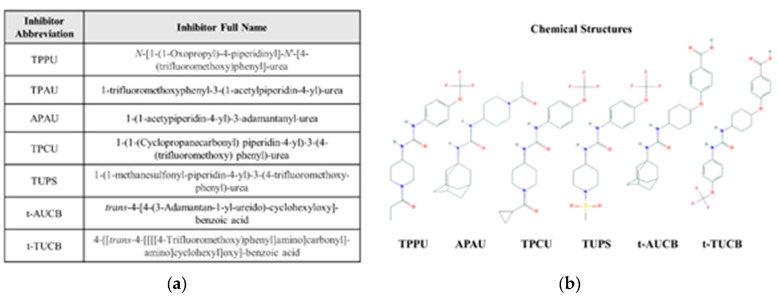
Commonly used soluble epoxide hydrolase inhibitors. (**a**) Abbreviations and full chemical names for commonly used inhibitors. (**b**) Chemical structures for representative inhibitors. Chemical structures were downloaded from the public PubChem database (pubchem.ncbi.nlm.nih.gov).

The potent biological effects of epFAs are abrogated due to their rapid hydrolysis by EHs—particularly sEH, the main EH responsible for endogenous lipid epoxide degradation [15]. sEH is a dual function enzyme with a C-terminal hydrolase domain and N-terminal phosphatase domain. The role of the phosphatase domain is not well characterized [16], but hydrolase domain-mediated breakdown of epFAs is a well-known process considered to deactivate epFAs. Indeed, dihydroxyeicosanoid products of epFAs (dihydroxyFAs) are generally less biologically active, inactive, or, in some cases, deleterious [17]. Logically, preservation of epFA levels by sEH inhibition is an attractive therapeutic option. To this end, numerous sEH inhibitors (sEHIs) have been developed using various pharmacophores, and with variable dissociation constants through the nanomolar and micromolar ranges. In the last decade, the disubstituted urea pharmacophore has gained popularity because of its higher potency than the previous generation of inhibitors [18]. Compounds in this class include TPPU, TPAU, APAU, TPCU, TUPS, AUCB, and t-TUCB (see Figure 2 for chemical structures and full chemical names); these inhibitors are used commonly in experimental animal and cell culture models of different pathologies. Indeed, preclinical animal models have demonstrated the efficacy of sEHIs in the treatment of atherosclerosis [19,20], kidney injury [21,22,23,24], acute lung injury [25,26,27,28], inflammatory bowel disease [29,30,31], angiogenesis and cancer [32,33], psychiatric and neurological disorders [34,35,36,37], sepsis [38], and more. Moreover, there are several clinical trials testing the effectiveness of sEHIs in humans. The sEHI GSK2256294 has completed a phase I clinical trial to determine its safety, pharmacokinetics, and efficacy in treating glucose intolerance (ClinicalTrials.gov NCT03486223) and smoking-related endothelial dysfunction [39]. Another compound, AR9281, has also completed phase I clinical trials for hypertension and insulin resistance treatment (ClinicalTrials.gov NCT00847899). Another sEHI, EC5026, is under development to reduce neuropathic pain in humans (NIH NIDA 1UG3DA048767-01).

There is accumulating evidence that sEH expression is induced in many liver pathologies, including non-alcoholic fatty liver disease (NAFLD), non-alcoholic steatohepatitis (NASH), liver fibrosis, and portal hypertension (PTH) [40,41,42,43,44,45]. While much effort has been expended to develop treatments, these liver diseases remain a global health burden. For example, global prevalence of NAFLD is estimated at approximately one in four [46]. Preservation of the remaining epFA pool by sEH inhibition represents an exciting novel therapeutic strategy with low adverse effects [47] to address this health crisis. In this review, we summarize the novel field of sEH inhibition in liver diseases by analyzing preclinical studies in several liver pathologies including NAFLD and associated metabolic disorders, NASH, and PTH. The reader is encouraged to consult Table 1 for categorical information on each key study reviewed.

## 2. Methodology

**Figure 3:**
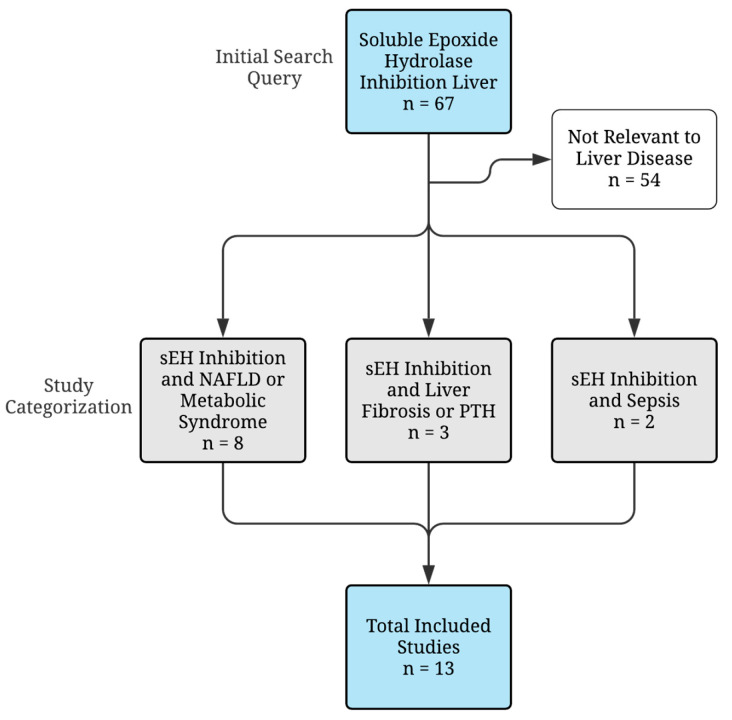
Flowchart describing the literature search strategy, exclusion criteria, and study categorization.

To provide a comprehensive review of the use of sEH inhibition in liver diseases, the PubMed database (https://www.ncbi.nlm.nih.gov/pmc/) was searched for key literature sources (those listed in Table 1) up to 1 May 2020 using a combination of medical subject heading terms and text keywords “soluble epoxide hydrolase inhibition liver” or “sEH inhibition liver”. All studies were screened individually by title and abstract, and studies that did not pertain to the liver disease were excluded. Date of publication, model organism, or sEHI used were not used to exclude liver studies. Our initial search yielded 67 studies, 54 of which were excluded for lack of relevance to liver disease. The remaining 13 studies were categorized by liver disease subtype (i.e., NAFLD/metabolic syndrome, fibrosis/PTH, and sepsis). Figure 3 describes the literature search strategy.

## 3. sEH Inhibition in Metabolic Syndrome and Non-Alcoholic Fatty Liver Disease

Epidemiological data show a strong association between NAFLD and metabolic syndrome, but the connection between the two is more than correlative: insulin resistance and lipid accumulation associated with metabolic disorder contribute directly to NAFLD pathogenesis [54]. For example, hepatic steatosis, an early manifestation of NAFLD, is the result of accumulation of “benign” lipid vesicles in the liver without significant inflammation or cell death and is common in metabolic syndrome patients [55]. From here, a minority of patients with NAFLD develop progressive NASH, wherein steatosis is accompanied by hepatic inflammation (primarily mediated by neutrophils and macrophages/recruited monocytes) and hepatocyte death [56]. NAFLD and NASH patients may or may not develop accompanying fibrosis, a complication that increases mortality [57]. However, the highest morbidity and mortality in non-alcoholic liver disease patients is not due to end-stage liver complications, but rather due to associated cardiovascular disease and cancer [58].

There are several pre-clinical studies demonstrating the beneficial effects of sEH inhibition in NAFLD and associated metabolic abnormalities. A report by Iyer et al. investigated the efficacy of sEH inhibition in metabolic syndrome using male Wistar rats fed a high-carbohydrate, high-fat (HCHF) diet ad libitum with or without the sEHI t-AUCB administered in the drinking water [48]. Compared to HCHF controls, HCHF rats treated with t-AUCB had significant improvements in metabolic endpoints including plasma lipid levels and insulin sensitivity. The study also investigated pathological changes related specifically to the liver, demonstrating that sEH inhibition by t-AUCB attenuated HCHF diet-induced liver hypertrophy, steatosis, and injury (confirmed by decreased lactate dehydrogenase and aspartate aminotransferase levels [LDH and AST, respectively], plasma biomarkers of liver injury). However, they reported no protection against HCHF diet-induced liver immune cell infiltration. This report on the pathogenic role of sEH in NAFLD has been corroborated by a host of other studies. For example, Liu et al. used an eight-week high-fat diet (HFD) feeding to induce NAFLD in C57BL/6 mice, then administered t-AUCB for four additional weeks (i.e., a ‘treatment’ paradigm) [40]. Whole-body sEH knockout (*Ephx2*^−/−^) mice were also fed a control or HFD. Compared to controls, HFD + t-AUCB mice had decreased steatosis as shown by liver hematoxylin and eosin (H&E) staining and reduced liver triglyceride content. Unlike Iyer et al., Liu et al. demonstrated a marked improvement in liver inflammation in HFD mice given t-AUCB or in *Ephx2*^−/−^ mice. Specifically, decreased macrophage accumulation as determined by F4/80 immunohistochemistry and decreased mRNA expression of numerous pro-inflammatory cytokines (e.g., TNFα, IL-6, MCP-1, and IFNγ) were observed. In this study, t-AUCB was also administered in a prevention paradigm, where the inhibitor was added to the drinking water prior to the animals being placed on a longer term 16-week HFD feeding protocol. In this paradigm, the NAFLD phenotype was similarly abrogated—decreased liver organ hypertrophy, decreased steatosis and liver/plasma triglycerides, and decreased inflammatory cytokine expression were reported in this model. Further, markers of inflammatory pathway activation (JNK and p38) were reduced. Conversely, when mice were injected with adenoviruses encoding human sEH, metabolic syndrome was exacerbated—liver/plasma lipids were increased, pro-inflammatory cytokine production was increased, and JNK and p38 proteins were increased. sEH inhibition or deletion also caused a reduction in HFD-induced adipose tissue inflammation, suggesting a role for the adipose-liver axis in sEH-mediated liver pathology. A study by Yao et al. also supports a pathogenic role for sEH in NAFLD [45]. Here, a high methionine diet (HMD) was used to induce hyperhomocysteinemia (HHcy) and hepatic steatosis. HHcy is prevalent in individuals with NAFLD and is considered a significant risk factor [59,60]. In mice fed an HMD, sEH inhibition by TPPU ameliorated hepatic steatosis as shown by liver H&E staining and decreased hepatic triglycerides compared to controls, likely due to an increase in the expression of β-oxidation genes (*Cpt1α*, *Acox1*, and *Mcad*).

Mechanistically, evidence generated by Sun et al. suggests that sEH inhibition may reduce hepatic inflammation by blocking inflammasome activation [43]. While the inflammasome is a key protein complex that regulates the adaptive response of the liver to pathogenic challenge, evidence also suggests a deleterious role in numerous liver diseases [61]. Sun et al. demonstrated that administration of 4-(5-phenyl-3-{3-[3-(4-trifluoromethyl-phenyl)-ureido]-propyl} *S*-pyrazol-1-yl) benzenesulfonamide (PTUPB) reduced inflammasome activation, as evidenced by a decrease in *Nlrp3*/NLRP3 and *Asc* expression, compared to HFD-fed mice without PTUPB. HFD-induced expression of downstream inflammasome targets pro-caspase 1, pro-IL1β, pro-IL18, and caspase 1 p10 were also decreased by PTUPB [43]. Additionally, administration of PTUPB reduced expression of hepatic pro-inflammatory cytokines TNFα, MCP1, and IL-6. It should be mentioned that PTUPB is a dual inhibitor of both sEH and cyclooxygenase 2, meaning future research may be needed to confirm the role of sEH specifically in inflammasome activation.

Hepatic endoplasmic reticulum (ER) stress is a known cellular consequence of NAFLD and metabolic syndrome. During ER stress, accumulation of unfolded proteins in the ER lumen activates the unfolded protein response (UPR), a signaling pathway which acts to either mitigate the burden of unfolded proteins or trigger apoptosis [62]. ER stress is both a consequence and a driver of liver disease, due to the UPR’s ability to trigger inflammation, inflammasome activation, and hepatocyte death [63]. Bettabieb et al. demonstrated a significant improvement in liver/adipose ER stress in *Ephx2*^−/−^ mice and wild-type (WT) mice administered TUPS [41]. Specifically, HFD-induced mRNA expression of BiP, the ER lumen unfolded protein ‘sensor’, was decreased by sEH inhibition or deletion, as was activation of PERK, IRE1α, and ATF6, the three ER membrane-bound proteins that activate each of the three branches of the UPR. Consequently, expression of downstream effectors of these three branches (e.g., XBP1 splicing, GADD34, and phopho-eIF2α) was also decreased at the protein level. Importantly, protein expression of the pro-apoptotic transcription factor CHOP, which lies downstream of all three branches of the UPR, was attenuated as well, indicating the pathogenic role of sEH in nearly every step of the UPR.

Lopez-Vicario et al. further corroborated the ability of sEH inhibition to attenuate hepatic steatosis and inflammation (e.g., decreased macrophage infiltration, chemokine and pro-inflammatory cytokine production, and increased expression of the pro-resolution cytokine IL10) [42]. The ER stress associated with NAFLD/metabolic syndrome was also attenuated by sEH inhibition in this study, as demonstrated by decreased protein levels of phosphorylated IRE1α and eIF2α (which lies downstream of PERK), thereby corroborating the protection against ER stress previously shown by Bettabieb et al. with a different sEHI. One potential contributor to this reduced ER stress is autophagy, a homeostatic process that selectively degrades damaged organelles by engulfing and targeting them for lysosomal degradation. Autophagy plays a critical role in liver health by degrading lipid droplets (lipophagy), glycogen granules (glycophagy), mitochondria and peroxisomes to regulate metabolism, and importantly, portions of stressed ER (reticulophagy/ER-phagy) [64,65]. In numerous liver diseases (including NAFLD/metabolic syndrome, ALD, viral hepatitis, hepatocellular carcinoma [HCC], and others), autophagy is dysregulated, leading to metabolic imbalance and an inability to eliminate damaged cellular components/organelles [66]. Lopez-Vicario et al. demonstrated that sEH inhibition rescued autophagy dysregulation in a HFD mouse model of NAFLD using the sEHI t-TUCB [42]. Specifically, protein expression of autophagy-related indicators Atg12-Atg5 (a protein complex) and LC3II were decreased with HFD feeding but significantly rescued with t-TUCB.

## 4. sEH Inhibition in Hepatic Fibrosis and Portal Hypertension

Liver fibrosis is a pathological feature of multiple liver diseases, including NASH, severe ALD, viral hepatitis, and cholestatic liver disease. Progressive fibrosis ultimately leads to liver cirrhosis and subsequent liver failure [67]. The scar tissue produced during hepatic fibrosis consists of abnormal extracellular matrix (ECM) components deposited by activated fibroblasts, typically hepatic stellate cells (HSCs)—although other fibroblasts may also play a role [68]. Importantly, fibrosis and inflammation are highly interconnected—many of the same cytokines that induce an inflammatory response (e.g., IL6, IL1β, and TGFβ) also activate HSCs to trigger liver fibrosis. Evidence also demonstrates that pro-inflammatory eicosanoids like prostaglandins and leukotrienes can promote liver fibrosis [69,70], whereas epFAs have shown protection against fibrosis in several organs [71]. However, studies investigating the role of epFAs and sEH in liver fibrosis are limited.

One such report by Harris et al. demonstrated the ability of sEH inhibition to improve carbon tetrachloride (CCl_4_)-induced liver fibrosis in mice given TPPU in the drinking water [51]. Specifically, quantitation of liver picrosirius red staining (which binds collagen) showed significant induction of fibrosis by CCl_4_ and reversal back to control levels by TPPU. More mechanistically, TPPU decreased expression of genes associated with HSC activation such as *Col1a2*, *Col3a1*, *Itag2* (integrin α2), and *Tsp2* (thrombospondin 2). TPPU also decreased both the mRNA expression and activity of matrix metalloproteases (MMPs), enzymes that positively correlate with fibrosis. CCl_4_ is also a potent inducer of liver inflammation and ER stress, both of which contribute to HSC activation. Harris et al. also showed that TPPU attenuated CCl_4_-induced markers of inflammation (*Cxcr4* and *Ccr2*), ER stress (phospho-PERK, phospho-IRE1α, *Atf6*, and *Chop*), and fibrosis (*Tgfβ1*), indicating that sEH inhibition may inhibit liver fibrosis both directly by acting on pro-fibrotic mediators and indirectly by acting on contributing factors. The use of an additional sEHI (t-TUCB) as well as *Ephx2*^−/−^ mice validated these results, suggesting a pathological role for sEH in liver fibrosis. Similar protection against CCl_4_-induced liver fibrosis and portal pressure were obtained by Zhang et al. with the sEHI t-TUCB [44]. Specifically, t-TUCB decreased CCl_4_-induced HSC activation markers (e.g., *Tgfβ1*, *αSMA* [alpha smooth muscle actin], collagens I and III, and MMPs) and markers of inflammation (*Il1β*, *Il6*, *Tnfα*, and *Nfκb)*. In addition, readouts of oxidative stress, another cellular consequence of CCl_4_ administration associated with increased liver fibrosis, showed that t-TUCB decreased CCl_4_-induced malondialdehyde formation and rescued the CCl_4_-mediated loss of superoxide dismutase and glutathione.

A study by Deng et al. aimed to determine whether sEH inhibition could also ameliorate PTH in rats [52]. PTH is a complication of liver fibrosis characterized by alterations in vascular tone and increased intrahepatic vascular resistance (IHVR) which increases portal pressure and blood flow, leading to varices, ascites, hepatomegaly, and other clinical presentations [72]. This question is also of pertinent research interest due to the known role of epFAs, particularly EETs, in promoting vasodilation by activating endothelial nitric oxide synthase (eNOS) [73,74]. Deng et al. used a CCl_4_ administration model of fibrosis and intrahepatic PTH which recapitulates the vascular dysfunction and structural abnormalities associated with IHVR [72]. t-TUCB administration significantly improved CCl_4_-induced hemodynamic deficits, significantly decreasing portal pressure, portal blood flow, and IHVR while non-significantly decreasing mean arterial pressure. In an in situ liver perfusion study to assess endothelial function, the same research group also showed t-TUCB rescued the impaired vasorelaxation caused by CCl_4_. This phenotype was accompanied by increased phospho-eNOS protein, nitric oxide levels, and decreased caveolin-1 protein (which reduces eNOS activity). In addition to improving hemodynamics and endothelial function, t-TUCB also ameliorated the underlying fibrosis/HSC activation and inflammation, as indicated by decreased αSMA and NFκB protein levels, respectively. Therefore, this study suggests the ability of sEH inhibition to protect against PTH both by directly improving endothelial function, and thereby hemodynamics, but also indirectly by improving the underlying fibrosis and inflammation.

## 5. sEH Inhibition in Sepsis Models

One immunological manifestation of severe liver disease is sepsis—a condition characterized by dysregulated response to inflammation following invasion of gut bacteria and associated bacterial products [75]. Like PTH, sepsis is a common a complication of cirrhosis. Cirrhosis patients are more susceptible to bacterial infection than the general population [76], and later-stage (decompensated) cirrhosis is associated with yet a higher risk [77]. Given the anti-inflammatory role of some epFAs, sEH is a logical target for treating the underlying inflammation associated with sepsis. To this end, Fife et al. investigated the ability of sEH inhibition to attenuate sepsis in a lipopolysaccharide (LPS) injection model with the sEHI AUDA administered by osmotic pump or by using *Ephx2*^−/−^ mice [53]. Despite the ability of sEHIs to attenuate inflammation in the previous NAFLD models [40,42,43], AUDA and genetic sEH deletion had limited ability to attenuate LPS-induced hepatic pro-inflammatory cytokine production (IL6, iNOS, TNFα) in the early phase of inflammation following LPS insult. By contrast, protection against LPS-induced inflammation has been demonstrated in a study by Schmelzer et al. where a higher LPS dose was used and analysis was performed at a later phase of inflammation following LPS administration [78].

A study by Chen et al. provides further evidence that sEHI-mediated protection against sepsis is model dependent by showing the beneficial effects of TPPU in a surgical cecal ligation and puncture (CLP) model [38]. This polymicrobial CLP sepsis model is arguably more comparable to human sepsis than LPS injection because it causes spillage of numerous fecal bacteria into the peritoneum, whereas LPS injection only introduces a single pathogen-associated molecular pattern [79]. Chen et al. showed mice that received the sEHI TPPU by oral gavage daily for five days prior to the procedure had a 20% improvement in CLP-induced mortality compared to vehicle controls (all control mice died within the 48-h period following the procedure). Additionally, TPPU improved signs of dysfunction in several organs, including the liver. Specifically, liver leukocyte infiltration and liver injury were significantly decreased compared to controls. Systemically, TPPU attenuated CLP-induced blood and peritoneum bacterial load and subsequently attenuated the inflammatory “cytokine storm” that follows, with decreases in systemic TNFα, IL1β, and IL6 levels following the surgery. Further, in a phagocytosis assay, TPPU increased RAW264.7 macrophage phagocytosis of fluorescent beads, indicating improved macrophage function. Collectively, these data suggest that sEH inhibition is a useful therapy in sepsis, but that model and phase of inflammation are critical factors determining its efficacy.

## 6. Molecular Mechanisms of sEHI-Mediated Protection against Liver Diseases

sEH may be responsible for the metabolism of some toxic xenobiotics, but the evidence reviewed here suggests a pathogenic role for sEH in liver diseases (Table 1). Indeed, many of the studies reviewed report an induction of sEH expression at the mRNA/protein level or an increase in sEH activity in different liver pathologies [40,41,42,43,44,45,52,53]. The efficacy of sEHIs generally lies in decreasing sEH activity, rather than decreasing expression of the gene/protein, although in some systems sEHIs do in fact modulate sEH protein levels. For example, Zhang et al. and Sun et al. reported that sEH inhibition by t-TUCB and PTUPB significantly reduced sEH protein in animals with PTH and NAFLD, respectively [43,44]. By contrast, Lopez-Vicario showed an increase in sEH protein in two different mouse genotypes after t-TUCB administration [42]. Translational curiosities aside, the protection against liver diseases afforded by sEHIs is likely due to preservation of epFAs which would otherwise be hydrolyzed by sEH. Indeed, the health benefits of epFAs derived from omega-3 and omega-6 PUFAs have long been demonstrated. In 1999, the anti-inflammatory properties of AA-derived EETs were discovered and linked to downregulation of NFκB [8]. EETs also promote organ regeneration/compensatory growth (including regeneration of the liver), wound healing, and retina/cornea vascularization [13]. EpFAs have also been implicated in preventing inflammation and promoting the resolution of inflammation [12]. While sEH inhibition is the more logical approach pharmacologically, boosting epFA pools by increasing their synthesis rather than preventing their degradation also improves disease phenotype. Evidence shows that liver overexpression of the epFA-producing *Cyp2j2* improves NAFLD endpoints like steatosis, inflammation, and oxidative stress [49].

In vitro studies suggest a link between individual epFAs and mechanisms underlying liver pathology such as lipid/cholesterol accumulation, insulin signaling, ER stress, autophagy, and inflammation. With respect to lipids and cholesterol, Mangels et al. showed a beneficial role for LA-derived 12,13-EpOME in cholesterol homeostasis in HepG2 cells [50]. In this study, 12,13-EpOME, but not 12,13-DiHOME, decreased protein expression of HMG CoA reductase, the rate limiting enzyme in cholesterol biosynthesis, by increasing expression of its inhibitor AMP-activated protein kinase. Lopez-Vicario et al. showed that palmitic acid (PA)-induced lipid accumulation in primary mouse hepatocytes was abrogated by AA-derived 14,15-EET, EPA-derived 17,18-EpETE, and DHA-derived 19,20-EpDPA [42]. EpFAs are also implicated in insulin signaling, another critical factor in metabolic syndrome and NAFLD. Bettabieb et al. treated HepG2 cells with unspecified EETs and EpOMEs, which enhanced basal and insulin-stimulated insulin receptor phosphorylation and protein kinase B phosphorylation (which induces glucose transport) [41]. With respect to ER stress, the same EETs and EpOMEs showed no effect on PA-induced ER stress, but interestingly, the sEH hydrolysis products (DiHETEs and DiHOMEs) exacerbated ER stress (as shown by phospho-IRE1α, phospho-eIF2α, and phospho-PERK levels) suggesting that sEHI-mediated protection may come in the form of epFA preservation or dihydroxyFA depletion [41]. Interestingly, Lopez-Vicario et al. were able to demonstrate attenuated ER stress with 14,15-EET, 17,18-EpETE, and 19,20-EpDPA in primary hepatocytes. These three epoxides also increased the LC3II:LC3I protein ratio, indicating enhanced activation of autophagy. With respect to inflammation, Chen et al. showed the anti-inflammatory properties of epFAs in the liver, demonstrating that 14,15-EET decreased PA-induced pro-inflammatory cytokine production (e.g., TNFα, IL6, and IL1β) in HepG2 cells [49]. This was accompanied by decreased NFκB signaling, consistent with previous studies connecting epFAs to NFκB blockade [8]. In addition to hepatocytes, cell culture studies also support a mechanistic role for epFAs in monocytes/macrophages. Chen et al. investigated 14,15-EET’s ability to alter cytokine release and phagocytosis in RAW264.7 murine macrophages. 14,15-EET had no effect on phagocytic ability when administered alone but did decrease LPS-induced expression of pro-inflammatory cytokines TNFα, IL1β, and IL6 [38]. By contrast, LA-derived 9,10-EpOME and 12,13-EpOME were shown to induce pro-inflammatory cytokine expression (*Il6* and *Mcp1*) in RAW264.7 cells in a dose-responsive manner [80]. Interestingly, sEH hydrolysis deactivated these LA-derived epFAs; corresponding dihyroxyFAs 9,10-DiHOME and 12,13-DiHOME did not influence *Il6* and *Mcp1* expression. These results suggest an immunomodulatory role for sEH in either preventing or permitting macrophage activation, depending on the epFA or dihydroxyFA considered.

Mechanistically, epFAs may act via several receptors, including peroxisome proliferator-activated receptors alpha and gamma (PPARα and PPARγ, respectively) [10,81]. These PPARs are considered therapeutic targets for the treatment of liver diseases like NAFLD due to their role in regulating glucose and lipid metabolism [82]. Subsequently, inhibition of PPARγ abrogates the beneficial effects of sEH inhibition in various diseases [83,84,85], and inhibition of PPARα abrogated the protective effect of TPPU on steatosis in mice in the study by Yao et al. [45]. At the level of individual epFAs, competition binding studies show that EETs (specifically 5,6-EET, 8,9-EET, 11,12-EET, and 14,15-EET) bind PPARγ with dissociation constants in the low micromolar range [81]. EPA-derived 17,18-EpEET was similarly shown to act through PPARγ to exert its anti-inflammatory effects [10]. Further, 11,12-EET, 14,15-EET, and 14,15-DiHETE, the hydrolysis product of 14,15-EET, were shown to activate PPARα in a luciferase reporter system, with 14,15-DiHETE activating PPARα at a level nearly three times higher than that of 14,15-EET, suggesting that epFAs and dihydroxyFAs may interact with nuclear receptors differently [45,86]. Some sEHIs are may even act as PPARα ligands themselves [87], suggesting that sEHI-mediated control of inflammation and lipid/glucose metabolism through PPAR receptors may be controlled both directly by the sEHI and indirectly by epFAs and dihydroxyFAs.

Another important point to consider is that there may be differences in the biological activities of epFAs based on the parent PUFAs. Data reviewed here suggest that omega-3-derived epFAs may have greater efficacy than omega-6-derived epFAs in blocking cellular characteristics of liver pathology. This idea is analogous to the more established concept that omega-3 PUFAs themselves are beneficial in liver disease, whereas omega-6 PUFAs are more pathogenic [88]. Specifically, Lopez-Vicario et al. showed that the omega-3 (EPA/DHA)-derived lipids included in their study (17,18-EpETE and 19,20-EpDPA) were more efficacious than the omega-6 (AA)-derived 14,15-EET in reducing PA-induced lipid accumulation/ER stress and promoting autophagy [42]. The differential effects of omega-3 vs. omega-6-PUFA-derived epFAs are often not directly considered; additional research in this area will likely address this question in the future.

## 7. Future Directions and Knowledge Gaps

While the initial discovery of epoxide hydrolases occurred approximately fifty years ago, the pathogenic role of sEH in liver diseases has been defined only recently. Clearly, sEH inhibition is an attractive therapeutic option in a spectrum of liver diseases, but future research in some key areas is needed to further elucidate its applications, limitations, and molecular mechanisms. One area that should be addressed is sex-specific responses to sEH inhibition in liver diseases. None of the studies reviewed here included female animals, despite the well-known sexual dimorphism of some liver diseases such as NAFLD in both humans and rodents [89]. Importantly, evidence suggests that estrogens can downregulate sEH expression, meaning females may have higher baseline epFA levels, affording some protection against liver disease, although no studies have addressed this question directly [90,91,92]. Future research should also address the efficacy of sEH inhibition in additional liver diseases like ALD, viral hepatitis, cholestatic liver disease, and HCC. These diseases share some characteristics with the liver diseases reviewed here (e.g., chronic inflammation, oxidative stress, and ER stress), characteristics which epFA preservation may attenuate [93,94,95]. Next, the pool of epFAs is quite a large one. However, the role and underlying mechanisms of only a few have been investigated so far. Future research should incorporate additional epoxides from multiple PUFAs to elucidate key mediators of liver diseases and to better understand whether omega-3 PUFAs truly do give rise to more beneficial epoxides than omega-6 PUFAs. Another important area to consider is that sEH is a dual-function enzyme with two bioactive domains—a C-terminal hydrolase domain (which sEHIs target) and an N-terminal phosphatase domain. While the hydrolase domain is well studied, the phosphatase domain is not, meaning that its potential contribution to liver pathology is unknown. Future studies should consider that the phosphatase activity remains active even after addition of sEHIs but is inactivated by genetic sEH deletion: this may help elucidate any differences in phenotype between sEHI-treated animals and *Ephx2*^−/−^ animals.

The ultimate goal of sEHI research in preclinical animal models is translation to human diseases. It is important to mention that humans have various polymorphisms in the *EPHX2* gene associated with increases or decreases in sEH activity [96,97]. Specifically, substitution of lysine 55 with arginine (Lys55Arg) is associated with higher hydrolase activity, whereas substitution of arginine 287 with glutamine (Arg387Gln) is associated with decreased hydrolase activity. These functional variants are associated with altered risk of cardiovascular disease and insulin sensitivity [98,99,100], and may contribute to heterogeneity in response to sEHI therapy between individuals. Clinical trials are nonetheless underway involving sEHIs for the treatment of insulin resistance, glucose intolerance, hypertension, endothelial dysfunction, and pain. Given the early success of these compounds, future clinical trials should help bring sEHIs into the market for liver-specific diseases in the coming years.

## 8. Conclusions

**Figure 4:**
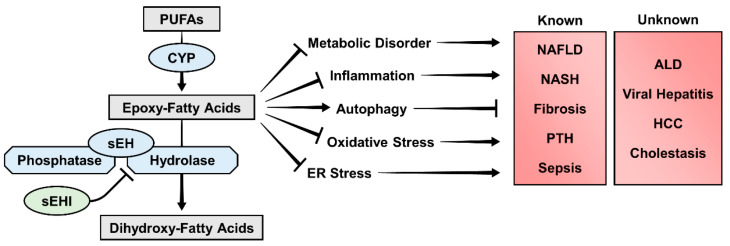
Summary figure. sEH inhibitors preserve levels of endogenously produced epFAs by preventing their sEH hydrolysis. sEH inhibition improves disease phenotype in non-alcoholic liver disease, non-alcoholic steatohepatitis, hepatic fibrosis, cirrhotic portal hypertension, and cirrhotic sepsis. Individual epFAs are shown to improve insulin resistance, inflammation, autophagy, oxidative stress, and endoplasmic reticulum stress, suggesting a mechanistic link between sEH inhibitors and protection against liver diseases. Future research should interrogate the efficacy of sEH inhibition in ALD, viral hepatitis, hepatocellular carcinoma, and cholestatic liver disease. PUFAs, polyunsaturated fatty acids; CYP, cytochrome P450 2J/2C families; sEH, soluble epoxide hydrolase; sEHI, soluble epoxide hydrolase inhibitor; NAFLD, non-alcoholic fatty liver disease; NASH, non-alcoholic steatohepatitis; PTH, portal hypertension; ALD, alcohol-associated liver disease; HCC, hepatocellular carcinoma.

sEH inhibition is a promising therapeutic strategy for the treatment of numerous liver diseases, including NAFLD. sEHIs show an efficacy in treating liver diseases that is typically not specific to one inhibitor nor one disease model. In vitro studies similarly show impressive efficacy of individual epFAs whose degradation is prevented by sEHIs in vivo, drawing a clear connection between sEH inhibition, epFAs, and attenuation of liver pathology (Figure 4). Continued research to evaluate sEH inhibition in additional liver diseases, sex differences, and molecular mechanisms should make translation of sEH inhibition to the clinic possible in the near future.

## Figures and Tables

**Table 1:** Summary of studies investigating the role of sEH in liver diseases. Listed are studies cited in the review, along with details of the experimental design (model, species, and sEHI used) and a brief description of the results and mechanism, if available. Changes in sEH expression or activity are noted in the results column, where available.

Authors [Ref]	Inhibitor	Disease	Model	Result	Molecular Mechanisms	
					In Vivo	In Vitro
Iyer et al. [48]	t-AUCB	NAFLD	HFHC Diet, Rat	↓ Insulin Resistance ↓ Hypertension ↓ Steatosis ↓ Liver Hypertrophy	↓ Cholesterol ↓ GTT Glucose AUC	N/A
Liu et al. [40]	t-AUCB	NAFLD	HFD, Mouse	↓ Steatosis ↑ sEH Activity	↓ Plasma Inflammatory Cytokines ↓ Adipose Macrophage Infiltration	N/A
Bettaieb et al. [41]	TUPS	NAFLD	HFD, Mouse	↓ Hepatic/Adipose ER Stress ↓ Cell Death, in vitro ↑ Insulin Signaling, in vitro ↑ sEH Protein	↓ BiP, XBP1, CHOP ↓ Caspase 3, cJUN, JNK, p38	EpOMEs and EETs in HepG2 cells: ↑ phospho-IR, phospho-AKT
Lopez-Vicario et al. [42]	t-TUCB	NAFLD	HFD, Mouse	↑ Brown Fat ↑ Hepatic Autophagy ↓ Steatosis ↑ sEH Protein	↑ IL10, RELMα, CD206, MGL1 ↑ M2 Polarization	14,15-EET, 19,20-EpDPA, and 17,18-EpETE in Primary Hepatocytes: ↓ Lipid accumulation ↓ phospho-eIF2α, phospho-IRE1α ↑ LC3II:LC3I ratio
Sun et al. [43]	PTUPB	NAFLD	HFD, Mouse	↓ Body/Liver Weight ↓ Liver Injury and Steatosis ↓ Fibrosis ↓ Inflammation ↑ sEH Protein	↓ NLRP3 Inflammasome Activation ↓ Inflammatory Cytokines ↓ COX2 Expression	N/A
Chen et al. [49]	N/A	NAFLD	HFD, Mouse	↓ Steatosis ↓ Inflammation ↓ Oxidative Stress	↓ NFκB ↓ JNK ↑ SOD, GPX	14,15-EET in HepG2 cells: ↓ NFκB, TNFα, IL1β, IL6 14,15-EET in RAW264.7 cells: ↓ TNFα, IL1β, IL6
Yao et al. [45]	TPPU	NAFLD	HMD, Mouse	↓ Steatosis ↑ sEH Protein	↑ Fatty Acid β-Oxidation Genes ↑ PPARα Activation	sEH Inhibition and 11,12-EET in Primary Hepatocytes: ↑ PPARα Activation
Mangels et al. [50]	t-AUCB	Metabolic Syndrome	Mouse	↓ Cholesterol	↑ AMPK Activation ↓ SREBP1 ↓ HMG CoA Reductase	12,13-EpOME in vitro: ↑ phospho-AMPK ↓ HMG CoA Reductase
Harris et al. [51]	TPPU	Liver Fibrosis	CCl_4_, Mouse	↓ Fibrosis ↓ ER Stress	↑ Metalloproteases ↓ Col1a2/Col3a1 mRNA ↓ JNK, Caspase 3	N/A
Zhang et al. [44]	t-TUCB	Liver Fibrosis	CCl_4_, Rat	↓ Fibrosis ↓ Portal Hypertension ↓ Inflammation ↓ Oxidative Stress ↑ sEH Protein	↓ TGFb ↓ Smad ↓ NFkB ↑ Metalloproteases ↑ SOD, GSH	N/A
Deng et al. [52]	t-TUCB	Portal Hypertension	CCl_4_, Rat	↓ Portal Pressure ↓ Liver Fibrosis ↓ Liver Endothelial Dysfunction ↑ sEH Protein	↑ p-eNOS ↑ NO ↓ Caveolin 1 ↓ NFkB	N/A
Fife et al. [53]	AUDA	Sepsis	LPS, Mouse	*n.s.* Inflammation ↑ sEH Activity by Lipidome	↓ iNOS	N/A
Chen et al. [38]	TPPU	Sepsis	Cecal Ligation, Puncture, Mouse	↑ Survival ↓ Organ Damage ↓ Systemic Inflammation	↑ MAPK Signaling ↑ Macrophage Phagocytosis ↓ Inflammatory Cytokines ↓ ALT/AST, BUN ↓ Bacterial CFU’s	14,15-EET in vitro: ↓ TNFα, IL1β, IL6 ↑ IL10
